# *ADRB2* polymorphisms predict the risk of myocardial infarction and coronary artery disease

**DOI:** 10.1590/S1415-475738420140234

**Published:** 2015

**Authors:** Dong-Wei Wang, Min Liu, Ping Wang, Xiang Zhan, Yu-Qing Liu, Luo-Sha Zhao

**Affiliations:** 1 Department of Cardiology, Zhengzhou Central Hospital Affiliated to Zhengzhou University, Zhengzhou, Henan, P.R. China; 2 Department of Cardiology, The First Affiliated Hospital of Zhengzhou University, Zhengzhou, Henan, P.R. China

**Keywords:** beta-2 adrenergic receptor, genetic polymorphism, myocardial infarction, coronary artery disease, meta-analysis

## Abstract

Recently, the rs1042713 G > A and rs1042714 C > G polymorphisms in the beta-2 adrenergic receptor (ADRB2) gene were shown to be related to atherosclerosis diseases. Therefore, we performed a systemic meta-analysis to determine whether the two functional polymorphisms are related to the risk of myocardial infarction (MI) and coronary artery disease (CAD). We identified published studies that are relevant to our topic of interest. Seven case-control studies, with a total of 6,843 subjects, were incorporated into the current meta-analysis. Our analysis showed a higher frequency of rs1042713 G > A variant in patients with MI or CAD compared to healthy controls. A similar result was also obtained with the rs1042714 C > G variant under both the allele and dominant models. Ethnicity-stratified subgroup analysis suggested that the rs1042714 C > G variant correlated with an increased risk of the two diseases in both Asians and Caucasians, while rs1042713 G > A only contributes to the risk of two diseases in Asians. In the disease type-stratified subgroups, the frequencies of both the rs1042713 G > A and rs1042714 C > G variants were higher in the cases than in the controls in both the MI and CAD subgroups. Collectively, our data contribute towards understanding the correlation between the rs1042713 G > A and rs1042714 C > G polymorphisms in *ADRB2* and the susceptibility to MI and CAD.

## Introduction

Coronary artery disease (CAD), the most common category of heart disease, is the leading cause of the hospital admissions, resulting in a high mortality in 2012 (Finegold *et al.*, 2013). CAD is induced by a plaque of fat, cholesterol and white blood cells that accumulate along the inner walls arteries of the heart, which narrows the arteries and reduces the rate and mass of blood flow to the heart (Korosoglou *et al.*, 2011). Myocardial infarction (MI), also referred to as acute myocardial infarction (AMI), accounts for the majority of the overall mortality in CAD (Korosoglou *et al.*, 2011). In 2010, over one million people in America experienced either their first or recurrent MI, and more than half of them died from it (Dupre *et al.*, 2012). During MI, patients gradually experience sudden chest pain beneath the thoracic cage and sometimes spreading to the left part of the neck or left arm. Additional symptoms include abnormal heartbeat, shortness of breath, feeling of indigestion, nausea or vomiting, sweating and anxiety (Kosuge *et al.*, 2006). The risk-related factors for MI include advanced age, a history of CAD, cigarette smoking, high serum concentrations of some lipids like triglycerides and low density lipoprotein cholesterol, decreased levels of high-density lipoprotein cholesterol, a lack of physical activity, heavy consumption of alcohol, intake of amphetamines and cocaine, and excess stress (Devlin and Henry, 2008; Graham *et al.*, 2007; Maclean, 2010). Genetic polymorphisms have recently been identified as an important risk factor in the pathology of CAD, including MI (Shea *et al.*, 2011; Tomaiuolo *et al.*, 2012).

The beta-2 adrenergic receptor (ADRB2) is a member of the superfamily of G-protein coupled receptors (GPCRs) (Cherezov *et al.*, 2007; Tchivileva *et al.*, 2010). The ADRB2 is widely expressed in most cell types, and it is the primary target of the catecholamine epinephrine during the stress response (Panebra *et al.*, 2010). ADRB2 signaling promotes cardiomyocyte survival and exerts sustained effects in the progenitor cells to regulate the differentiation, proliferation and mobility of the cells (Khan *et al.*, 2013). The *ADRB2* gene is located on the long arm of chromosome 5q31-q32. Structurally, it is an intronless gene that encodes a 413 amino acid protein product (Neuman *et al.*, 1992; Ortega *et al.*, 2014). In recent years, several genetic polymorphisms have been identified in *ADRB2*, including rs1042713 G > A and rs1042714 C > G, and various studies have concentrated on the associations between these genetic polymorphisms and cardiovascular diseases (Li *et al.*, 2013; Zak *et al.*, 2008). *ADRB2* polymorphisms are relevant to several types of cardiovascular diseases, such as hypertension, heart failure, MI and CAD (Brodde, 2008; Kulminski *et al.*, 2010; Lou *et al.*, 2010). ADRB2 activation regulates various biological functions, including the heart rate, blood pressure or respiration, and it may modulate the vasodilatation of the microcirculation in normal coronary arteries (Barbato *et al.*, 2005). The ADRB2 plays an important modulatory role in the vasodilatation of human coronary arteries, and *ADRB2* polymorphisms have been reported to alter the functional responses of the receptor, which may lead to increased vasodilation and susceptibility to CAD (Barbato *et al.*, 2007). In addition, a previous study showed that *ADRB2* polymorphisms might elevate sympathetic nerve activity, which is associated with the increased risk of MI (Schurks *et al.*, 2009). On the one hand, there is abundant evidence supporting the notion that *ADRB2* polymorphisms correlate with an increased risk of MI and CAD (Barbato *et al.*, 2007; Jia *et al.*, 2010). On the other hand, some important studies report contrary results (Sala *et al.*, 2001; Wallerstedt *et al.*, 2005). The current meta-analysis reported herein used carefully selected and reliable data from published studies investigating the role of *ADRB2* polymorphisms in MI and CAD development.

## Materials and Methods

### Data sources and eligibility criteria

To identify all pertinent papers that assessed the correlations of ADRB2 genetic polymorphisms with the susceptibility for MI and CAD, we comprehensively searched the PubMed, Embase, Web of Science, Cochrane Library, CINAHL, CBM and CNKI databases (last updated search in May 31^st^, 2014), utilizing selected common keywords for the ADRB2 gene, polymorphism, MI and CAD. The following keywords were applied in our literature search: (“receptors, adrenergic, beta-2” or “receptors, adrenergic, beta-1” or “receptors, adrenergic, beta” or “adrenergic beta-2 receptors” or “beta 2 adrenergic receptor” or “beta-2 adrenergic receptor” or “beta2AR” or “ADRB2” or “beta2-AR” or “adrenergic beta-1 receptors” or “beta 1 adrenergic receptor” or “beta-1 adrenergic receptor” or “beta1AR” or “ADRB1” or “beta1-AR”) and (“polymorphism, genetic” or “polymorphism” or “polymorphisms” or “variants” or “SNP” or “mutation” or “genetic variants”) for the exposure factors, as well as (“MI” or “coronary artery disease” or “CAD” or “MI” or “myocardial infarct” or “myocardial infarction” or “myocardium infarction” or “cardiac infarction” or “myocardia infarction” or “infarction myocardium” or “myocardial infarcted” or “heart infarction” or “heart infarction” or “MI” or “acute MI” or “CAD” or “CHD” or “AMI”). No restriction was set on the language of the article. We further scanned the bibliographies of the relevant articles manually to identify additional relevant papers. When the enrolled papers contained unclear or additional data in their original publications, the first authors were contacted and asked for clarification.

To enroll high-quality articles into the current meta-analysis, we searched case-control studies on genotypic data for *ADRB2* polymorphisms with human subjects with and without MI, or with and without CAD, that reported adjusted odd ratios (ORs) and 95% confidence intervals (CI). We only extracted studies that provided the sample number and sufficient information about the ADRB2 variants, and we excluded articles with incomplete, unavailable or inappropriate data, as well as those studies in which MI and CAD were not confirmed by histopathologic examinations. In addition, only studies with a minimum of 100 cases were selected for the meta-analysis. All selected studies were consistent with Hardy-Weinberg equilibrium (HWE) in the control group. When 50% of the subjects in the extracted studies overlapped in more than two papers, we enrolled the most comprehensive study. Only the newest or most complete study was included when the same authors or group published the extracted studies.

### Study selection

Initially, a total of 243 articles were retrieved. During study selection, the titles and abstracts of the retrieved studies were screened based on the eligibility criteria detailed above, and 106 of the studies were excluded. Subsequently, the full texts of the remaining studies were carefully reviewed, and 103 studies failed to meet the eligibility criteria. Any ambiguities or disagreements on the eligibility for our meta-analysis were discussed to reach a final consensus among several reviewers. After stringent study selection, seven high-quality case-control studies were enrolled in the final analysis (Sala *et al.*, 2001; Wallerstedt *et al.*, 2005; Zee *et al.*, 2005; Abu-Amero *et al.*, 2006; Barbato *et al.*, 2007; Jia *et al.*, 2010; Yilmaz *et al.*, 2009). The studies had been conducted in China and Turkey (representing Asian populations), as well as in Belgium, Saudi Arabia, USA, Sweden and Italy (representing Caucasian populations). The sources of controls in our present meta-analysis were from population-based (PB) subjects. The genotyping methods detecting *ADRB2* polymorphisms included in this meta-analysis were TaqMan and polymerase chain reaction-restriction fragment length polymorphism (PCR-RFLP) analyses, and the ADRB2 SNPs were rs1042714 C > G and rs1042713 G > A. All included studies, published between 2001 and 2010, were consistent with HWE (all p > 0.05). The baseline characteristics of the extracted studies are presented in Table 1.

**Table 1:** Baseline characteristics of the studies included in the present meta-analysis.

First author	Year	Disease	Country	Sample size		Gender (M/F)		Age (years)		Genotyping methods	SNP	STROBE
				Case	Control	Case	Control	Case	Control			Score
Jia LX	2010	CAD	China	428	397	317/111	254/143	56 ± 10.6	53.2 ± 10.5	PCR-LDR	rs1042713 G > A	35
Yilmaz AK	2009	MI	Turkey	100	100	82/18	56/44	54.2 ± 11.9	51.4 ± 11.6	PCR-RFLP	rs1042713 G > A, rs1042714 C > G	23
Barbato E	2007	CAD	Belgium	570	216	399/171	110/106	65.0 ± 10.0	60.0 ± 13.0	TaqMan assay	rs1042713 G > A, rs1042714 C > G	36
Abu-Amero	2006	CAD	Saudi Arabia	773	895	477/296	519/376	53.8 ± 1.08	50.5 ± 3.6	PCR-CTPP	rs1042714 C > G	38
Zee RY	2005	MI	USA	523	2092	–	–	58.7 ± 0.4	58.8 ± 0.2	TaqMan assay	rs1042713 G > A, rs1042714 C > G	30
Wallerstedt SM	2005	MI	Sweden	174	342	129/52	253/89	57.0 ± 6.6	57.1 ± 6.6	TaqMan assay	rs1042713 G > A, rs1042714 C > G	28
Sala G	2001	MI	Italy	125	108	125/0	108/0	45	45	PCR-RFLP	rs1042713 G > A, rs1042714 C > G	27

M: male. F: female. PCR-LDR: polymerase chain reaction-ligase detection reaction. PCR-RFLP: polymerase chain reaction-restriction fragment length polymorphism. PCR-CTPP: polymerase chain reaction-confronting two-pair primers. SNP: Single nucleotide polymorphism. STROBE: Strengthening the Reporting of Observational Studies in Epidemiology. MI: myocardial infarction. CAD: coronary artery disease.

### Data extraction

To reduce a potential bias and enhance reliability, two investigators independently extracted information from the retrieved papers according to the selection criteria and, through discussion and reexamination, reached consensus on all items. The following relevant data were prospectively extracted from the eligible studies for final analysis: surname of the first author, year of publication, source of publication, study type, study design, sample size, age, sex, ethnicity and country of origin, genotyping method, source of controls, disease type, available genotype, genotype and variant frequencies, and HWE evidence in controls. All authors agreed to and approved the final selection of the studies that were included in the analysis.

### Quality assessment

The pairs of investigators involved in data extraction used the Strengthening the Reporting of Observational studies in Epidemiology (STROBE) quality score systems to independently assess the studies for quality (Vandenbroucke *et al.*, 2014). STROBE comprised 40 assessment items associated with the quality appraisal, with scores ranging from 0 to 40. According to the STROBE scores, the included studies were classified into the following three levels: low quality (0-19), moderate quality (20-29), and high quality (30-40), respectively. Any discrepancies, if present, with the STROBE scores of the enrolled publications were resolved by discussion with a third reviewer. The methodological quality of the extracted studies is also presented in Table 1.

### Statistical analysis

The OR was one measure of interest for assessing the relationship of the ADRB2 variants with MI and CAD. However, the OR value is influenced by sample size and/or differences in ethnic background. Theoretically, if there was no significant difference in the baseline data, the OR values could be directly used in our meta-analysis; otherwise, a pooled ORs (summary ORs) estimate was chosen to enhance stability of the final value. To calculate the effect size for each study, the summary ORs with the 95%CI were computed with the *Z* test. To provide quantitative evidence for all selected studies and minimize the variance of the summary ORs with the 95%CI, we conducted the current statistical meta-analyses with a random-effects model (DerSimonian and Laird method) or fixed-effects model (Mantel-Haenszel method) of the individual study results, under the situation in which data from independent studies could be combined. The random-effect model was applied when there was heterogeneity among the studies, while the fixed-effects model was applied when there was no statistical heterogeneity. The subgroup meta-analyses were also conducted according to ethnicity, disease type and genotyping method, so as to explore the potential effect modification, and the heterogeneity across the enrolled studies was evaluated with the Cochran's *Q*-statistic (p < 0.05 was considered statistically significant) (Jackson *et al.*, 2012). As a result of the low statistical power of the Cochran's *Q*-statistic, the *I* ^2^ test was also measured to reflect the possibility of the heterogeneity between studies (Peters *et al.*, 2006). The *I* ^2^ test values ranged from 0% (no heterogeneity) to 100% (maximal heterogeneity). We utilized univariate meta-regression analysis and multivariate meta-regression analysis to evaluate the possible sources of heterogeneity, and further multiple calibration tests were conducted using the Monte Carlo method. One-way sensitivity analysis was performed to evaluate whether the results could have been significantly affected. This was done through deleting a single study in our meta-analysis, one by one, to evaluate the influence of an individual data set on the pooled ORs. A funnel plot was constructed to assess the publication bias, which might affect the validity of the estimates. The symmetry of the funnel plot was further evaluated by Egger's linear regression test (Zintzaras and Ioannidis, 2005). All tests were two-sided, and a p value of <0.05 was considered statistically significant. STATA software, version 12.0 (Stata Corp, College Station, TX, USA) was used to ascertain the credibility and accuracy of these results.

## Results

### Association of *ADRB2* polymorphisms with MI and CAD

**Figure 1:**
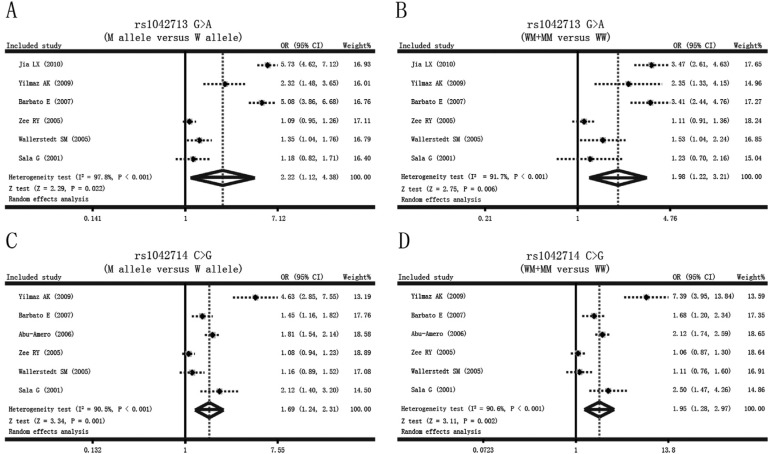
Forest plots of the influences of the *ADRB2* genetic polymorphism on the risk of myocardial infarction and coronary artery disease under the allele and dominant models.

As shown in Figure 1, the major findings of the present meta-analysis included a higher frequency of the rs1042713 G > A variant in the ADRB2 of patients with MI or CAD compared to healthy controls (allele model: OR = 2.22, 95%CI: 1.12-4.38, p = 0.022; dominant model: OR = 1.98, 95%CI: 1.22-3.21, p = 0.006). At the same time, the results in Figure 1 suggested a positive association of the ADRB2 rs1042714 C > G variant with the occurrence of MI or CAD (allele model: OR = 1.69, 95%CI: 1.24-2.31, p = 0.001; dominant model: OR = 1.95, 95%CI: 1.28-2.97, p = 0.002).

**Figure 2:**
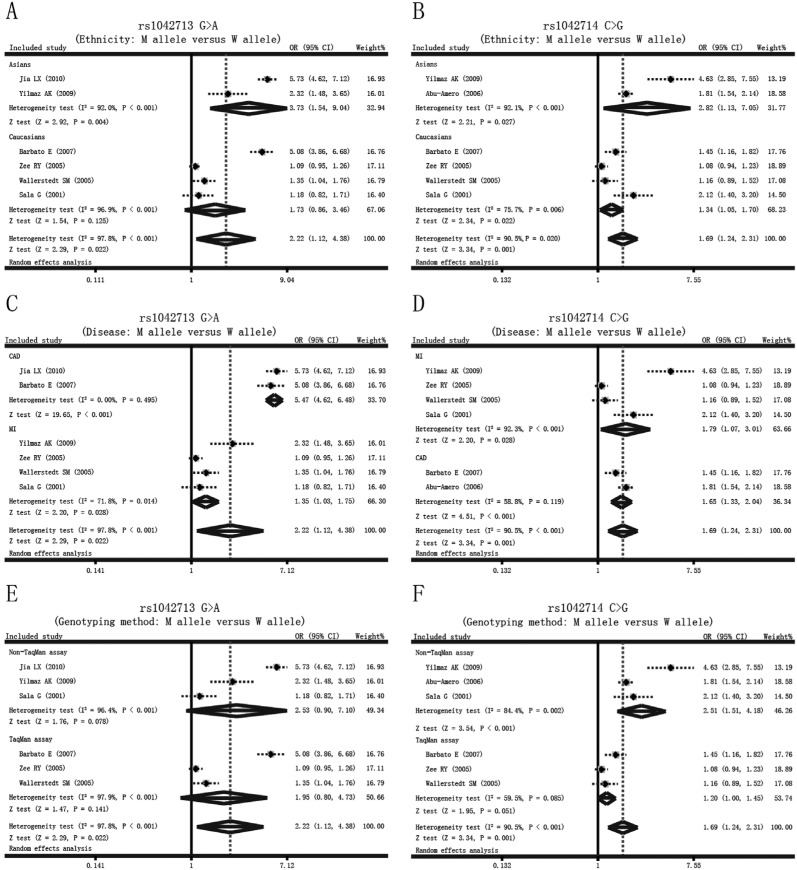
Subgroup analyses for the influences of the *ADRB2* genetic polymorphism on the risk of myocardial infarction and coronary artery disease under the allele model.

We observed differences in the association of rs1042713 G > A and rs1042714 C > G polymorphisms with MI or CAD among different ethnicities, disease types and genotyping methods, and further *Q*-test analysis revealed the presence of heterogeneity (*I* ^2^ > 90.5%, p < 0.05). Therefore, we conducted subgroup analyses. The subgroup analysis based on ethnicity showed that the rs1042714 C > G polymorphism in the ADRB2 was positively correlated to the risk of MI and CAD in both Asians and Caucasians (all p < 0.05) (Figure 2). However, the subgroup analysis by ethnicity (Figure 2) showed a positive correlation between the *ADRB2* rs1042713 G > A variant and MI or CAD in Asians (allele model: OR = 3.73, 95%CI: 1.54-9.04, p = 0.004), which was not the case for Caucasians (p = 0.125). Simultaneously, subgroup analyses by disease type revealed that the frequencies of the *ADRB2* rs1042713 G > A and rs1042714 C > G polymorphisms were higher in the case groups than in the control groups in both the MI and CAD subgroups (all p < 0.05) (Figure 2). A further subgroup analysis based on the genotyping method revealed that the rs1042714 C > G polymorphism in the *ADRB2* was positively correlated with MI and CAD in studies using Non-TaqMan assays (allele model: OR = 2.51, 95%CI: 1.51-4.18, p < 0.001) instead of the TaqMan assay (p = 0.051) (Figure 2). This subgroup analysis also revealed that the positive relationship with the *ADRB2* rs1042713 G > A variant was not associated with the susceptibility to MI or CAD, neither in the TaqMan, nor in the Non-TaqMan assay subgroup (both p > 0.05). The ethnicity, disease type and genotyping method subgroup analyses under the other four models (dominant model, recessive model, homozygous model and heterozygous model) are shown in Table 2. Additionally, univariate meta-regression and multivariate meta-regression analyses demonstrated that the publication year, ethnicities, disease types and genotyping methods were not the main sources of heterogeneity among the included studies, and they were not the key factors influencing the overall results (all p > 0.05), as shown in Table 3.

**Table 2:** Meta-analysis of the correlations of *ADRB2* genetic polymorphisms with myocardial infarction and coronary artery disease.

Subgroup analysis	M allele *vs.* W (Allele model)			WM + MM *vs.* WW (Dominant model)			MM *vs.* WW + WM (Recessive model)			MM *vs.* WW (Homozygous model)			MM *vs.* WM (Heterozygous model)		
	OR	95%CI	p	OR	95%CI	p	OR	95%CI	p	OR	95%CI	p	OR	95%CI	p
rs1042713 G > A	2.22	1.12-4.38	0.022	1.98	1.22-3.21	0.006	4.31	1.26-14.75	0.020	4.75	1.39-16.22	0.013	3.93	1.12-13.71	0.032
Ethnicity															
Asians	3.73	1.54-9.04	0.004	3.10	2.19-4.39	< 0.001	18.14	5.98-55.05	< 0.001	22.15	11.63-42.15	< 0.001	14.98	3.06-73.37	0.001
Caucasians	1.73	0.86-3.46	0.125	1.64	0.94-2.87	0.080	2.38	0.83-6.82	0.107	2.63	0.87-7.90	0.085	2.18	0.77-6.16	0.140
Disease																	
CAD	5.47	4.62-6.48	< 0.001	3.45	2.77-4.28	< 0.001	21.71	13.35-35.32	< 0.001	21.51	14.14-32.74	< 0.001	22.02	11.89-40.78	< 0.001
MI	1.35	1.03-1.75	0.028	1.41	1.04-1.91	0.028	1.43	0.93-2.18	0.101	1.72	1.00-2.93	0.048	1.22	0.88-1.70	0.235
Genotyping method															
Non-TaqMan assay	2.53	0.90-7.10	0.078	2.23	1.20-4.15	0.011	6.63	0.77-57.46	0.086	7.38	0.98-55.45	0.052	5.99	0.63-57.13	0.120
TaqMan assay	1.95	0.80-4.73	0.141	1.78	0.90-3.56	0.100	2.86	0.73-11.14	0.131	3.14	0.77-12.82	0.110	2.60	0.68-10.01	0.164
rs1042714 C > G	1.69	1.24-2.31	0.001	1.95	1.28-2.97	0.002	1.62	1.21-2.17	0.001	2.20	1.39-3.49	0.001	2.20	1.39-3.49	0.001
Ethnicity																
Asians	2.82	1.13-7.05	0.027	3.82	1.13-12.93	0.031	2.50	1.08-5.76	0.0.32	4.47	1.12-17.83	0.034	4.47	1.12-17.83	0.034
Caucasians	1.34	1.05-1.70	0.020	1.42	1.01-2.00	0.045	1.39	1.08-1.77	0.009	1.69	1.12-2.55	0.013	1.69	1.12-2.55	0.013
Disease																
MI	1.79	1.07-3.01	0.028	2.06	1.01-4.20	0.048	1.68	1.04-2.70	0.034	2.32	1.11-4.86	0.025	2.32	1.11-4.86	0.025
CAD	1.65	1.33-2.04	< 0.001	1.96	1.58-2.44	< 0.001	1.74	1.26-2.40	0.001	2.29	1.63-3.23	< 0.001	2.29	1.63-3.32	< 0.001
Genotyping method															
Non-TaqMan assay	2.51	1.51-4.18	< 0.001	3.24	1.65-6.35	0.001	2.16	1.43-3.27	< 0.001	3.67	1.75-7.69	0.001	3.67	1.75-7.69	0.001
TaqMan assay	1.20	1.00-1.45	0.051	1.24	0.93-1.64	0.142	1.32	1.05-1.66	0.020	1.51	1.02-2.23	0.040	1.51	1.02-2.23	0.040

W: wild-type allele. M: mutant allele. WW: wild-type homozygote. WM: heterozygote. MM: mutant homozygote. OR: odds ratio. 95%CI: 95% confidence interval. MI: myocardial infarction. CAD: coronary artery disease.

**Table 3:** Univariate and multivariate meta-regression analyses of potential source of heterogeneity.

Heterogeneity factors	rs1042713 G > A						rs1042714 C > G					
	Coefficient	SE	t	p	95%CI		Coefficient	SE	t	p	95%CI	
					LL	UL					LL	UL
Publication year												
Univariate	0.192	0.092	2.08	0.213	-0.064	0.449	0.242	0.218	1.11	0.059	-0.363	0.847
Multivariate	0.899	0.264	3.40	0.227	-2.458	4.257	0.636	0.059	10.82	0.051	-0.111	1.382
Ethnicity												
Univariate	0.750	0.750	1.00	0.211	-1.332	2.832	1.744	0.931	1.87	0.117	-0.840	4.328
Multivariate	-6.451	1.988	-3.25	0.232	-31.706	18.805	-2.565	0.360	-7.13	0.218	-7.136	2.006
Disease													
Univariate	1.604	0.260	6.17	0.936	0.882	2.326	-0.599	1.241	-0.48	0.126	-4.045	2.846
Multivariate	0.046	0.459	0.10	0.988	-5.789	5.881	-0.913	0.131	-6.95	0.225	-2.581	0.755
Genotyping method												
Univariate	0.182	0.784	0.23	0.191	-1.996	2.359	1.605	0.893	1.80	0.059	-0.874	4.084
Multivariate	3.587	1.086	3.30	0.232	-10.213	17.388	3.563	0.332	10.73	0.051	-0.655	7.781

SE: standard error. 95%CI: 95% confidence interval. UL: upper limit. LL: lower limit.

### Sensitivity analysis and publication bias

**Figure 3:**
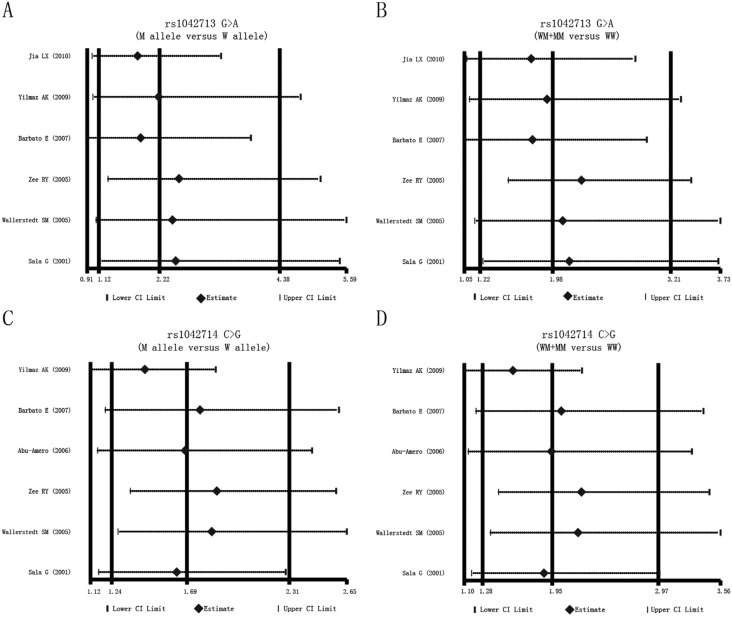
Sensitivity analysis for the influences of the *ADRB2* genetic polymorphism on the risk of myocardial infarction and coronary artery disease under the allele and dominant models.

**Figure 4:**
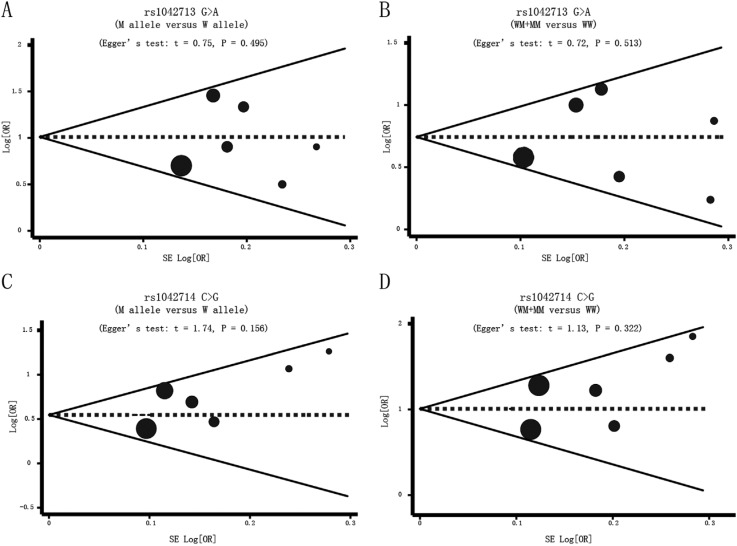
Funnel plot of publication biases on the relationships between the *ADRB2* genetic polymorphisms and the risk of myocardial infarction and coronary artery disease under the allele and dominant models.

A sensitivity analysis was performed to evaluate whether the present meta-analysis was stable. Each study enrolled in our meta-analysis was individually evaluated for its effect on the pooled ORs. The overall statistical significance did not change when any single study was omitted. Therefore, the current meta-analysis data are relatively stable and credible (Figure 3). The graphical funnel plots of the seven studies for the ADRB2 rs1042713 G > A and rs1042714 C > G variants were symmetrical, and Egger's test showed that there was no publication bias (all p > 0.05) (Figure 4).

## Discussion

In our meta-analysis on correlations between the polymorphisms of rs1042713 (R16G) and rs1042714 (Q27E) in the *ADRB2* with the susceptibility to MI and CAD based on available data, we found that the rs1042713 and rs1042714 polymorphisms are significantly associated with the susceptibility to MI and CAD. With its seven transmembrane segments, ADRB2 belongs to the superfamily of G-protein-coupled adrenergic receptors, and it is an important target of endogenous ligands, such as catecholamine and epinephrine, that mediate stress responses in humans and animals (Schurks *et al.*, 2009; Yilmaz *et al.*, 2009; Litonjua *et al.*, 2010).

The ARDB2 signaling cascade is of relevance in cardiovascular and metabolic diseases, including obesity, and also in mental disorders and asthma (Kushnir *et al.*, 2013). Additionally, accumulating evidence suggests that the ADRB2 could participate in astrocyte homeostasis and neuroprotection through the metabolism of glycogen, immune response regulation, and neurotrophic factor release in response to neuronal injury. Conversely, ADRB2 dysregulation may contribute to the development of Alzheimer's disease, stroke and hepatic encephalopathy (Laureys *et al.*, 2010). Furthermore, ADRB2 signaling is involved in bronchoprotection and bronchodilation through mucociliary clearance, the accumulation of fluid and basophilic mediator release, all of which play essential roles in the development of asthma (Hizawa, 2009).

The development of cardiovascular diseases, such as MI and CAD, is thought to involve ADRB2 through regulating the sympathetic and parasympathetic heart system influence on contractility and heart rate (Abu-Amero *et al.*, 2006). Moreover, the ADRB2 could reduce atherosclerotic plaque cellularity through reducing vascular smooth muscle cell proliferation, an important feature of atherosclerotic lesion formation, leading to instability and rupture of the plaques and increasing the risk of MI and CAD (Piscione *et al.*, 2008). The ADRB2 could also affect the vasodilatory function of vascular smooth muscle cells, leading to vasodilation and influencing the function and reactivity of cardiovascular cells (Wallerstedt *et al.*, 2005).

The two *ADRB2* polymorphisms, rs1042713 and rs1042714, are common in human populations and could lead to receptor alterations, affecting normal ADRB activity (Sala *et al.*, 2001). The rs1042713 and rs1042714 polymorphisms might also be related to agonists promoting desensitization and affecting hemodynamics and cardiac function (Cotarlan *et al.*, 2013). It has been reported that variants of the A/G site in rs1042713 have a strong relationship with CAD pathogenesis, and, in the dominant mode analysis, the low frequency of the A site in rs1042713 appears to be a key factor in CAD protection (http://www.cqvip.com/qk/93060a/201008/35012429.html). Abu-Amero *et al.* (2006) evaluated a Saudi Arabian sample and reported that the rs1042713 polymorphism may be an independent predictor of severe CAD, which is consistent with the findings of our meta-analysis. In addition, the Glu variant, compared to Gln in rs1042714, has been linked with vasodilatory responses to isoproterenol, which might be associated with atherosclerosis in cardiovascular diseases. One explanation could be that the Gln variant of rs1042714 results in a receptor with hyperactivity, leading to over-stimulation of catecholamine and over-activity of sympathetic nerves and, consequently, accelerating the development of coronary atherosclerosis (Barbato *et al.*, 2007). Zee *et al.* (2005), based on a US sample, reported that both of rs1042713 and rs1042714 polymorphisms are correlated with the development and progression of MI, which is also in line with our findings. Additionally, Heckbert *et al.* (2003) reported a possible relationship between the rs1042713 and rs1042714 polymorphisms in the *ADRB2* and a high risk of cardiovascular disease in the older age groups.

A stratified analysis, based on ethnicity and different disease types and detection methods, was performed to study the other influencing factors. A subgroup analysis based on ethnicity further showed that there were significant correlations between the rs1042713 and rs1042714 polymorphisms and the risk of MI and CAD. Our results are in agreement with other studies indicating that the rs1042713 and rs1042714 polymorphisms in the *ADRB2* gene have an intimate relationship with CAD and MI. Hence, the *ADRB2* polymorphisms might be an important contributor to cardiovascular diseases, as well as an important genetic marker for the diagnosis and prognosis of cardiovascular diseases.

Our study has some limitations. First, a study performed in a Saudi Arabian population was included in the present meta-analysis, and the results of that study were in agreement with our overall results. However, Saudi Arabia is a multi-racial population, which may influence the validity of the overall results. Second, very few epidemiological studies have explored how the ADRB2 gene is related to the susceptibility to MI or CAD, and most of the evidence that we gathered was from published composite coronary artery disease endpoints, including stroke, MI or CAD. This methodology may have restricted the extracted data. Third, all included studies had a case-control design; however, there were at least two apparent limitations. The sample size was relatively small, and the designed case-control studies always precluded causality. Therefore, it was difficult to reach a definitive conclusion. Fourth, with respect to the stratified analysis, there was a limitation in the subgroup analyses (ethnicity, disease, and genotyping method), and there was significant heterogeneity in some subgroups, restricting the overall interpretation of the pooled risk estimation. Finally, we only analyzed two ADRB2 variants, excluding the potential influence of other variants within the pathway.

Despite the aforementioned limitations, our findings support that the rs1042713 and rs1042714 polymorphisms of the ADRB2 gene have a strong correlation with, MI and CAD, when tested under both the allele and dominant models, particularly among Asians. This meta-analysis might serve as an anchoring point for designing further studies and developing ADRB2-based strategies to assess MI and CAD susceptibility.
